# Right Lower Pulmonary Vein Thrombi and Right Upper Pulmonary Vein Thrombi Connected on the Anterior Wall of the Left Atrium

**DOI:** 10.7759/cureus.62959

**Published:** 2024-06-23

**Authors:** Hidekazu Takeuchi

**Affiliations:** 1 Internal Medicine (Cardiology), Takeuchi Naika Clinic, Ogachi-Gun, JPN

**Keywords:** 80-mdct, tee, la thrombi, pulmonary vein, calcification of thrombi, la wall, rlpv, rupv

## Abstract

The interaction between the right upper pulmonary vein (RUPV) and the right lower pulmonary vein (RLPV) is poorly understood. In this paper, using transesophageal echocardiography (TEE) and 80-slice multidetector computed tomography (80-MDCT), we report that the RUPV thrombi and the RLPV thrombi invade the left atrium (LA) and reach the anterior wall of the LA. To our knowledge, this is the first study to directly show the connection between the RUPV thrombi and the RLPV thrombi on the anterior wall of the LA using TEE and 80-MDCT.

## Introduction

Research on thrombi retrieved from patients with ischemic stroke (IS) and acute myocardial infarction (AMI) has shown that the retrieved thrombi are calcified in some patients [1-4], which means that the retrieved thrombi are old. The retrieved thrombi existed before the occurrence of the IS and AMI. We reported that pulmonary vein thrombi (PVTs) are common [5,6] and can cause IS and AMI through the emission of large particles [7,8]. Understanding the characteristics of PVTs is important for preventing IS and AMI. We reported several cases of PVTs using cardiac computed tomography (CT) and transesophageal echocardiography (TEE) [9-13]. PVTs can release several sizes of particles. Large particles can cause IS and AMI, and small particles such as neutrophil extracellular traps (NETs) can be associated with acute coronary syndrome [14], heart failure [15], and type 2 diabetes mellitus (T2DM) [16]. Additionally, we reported that in many patients, PVTs form a network of PVTs in the left atrium (LA) [17], which was estimated using 64-slice multidetector computed tomography (64-MDCT). However, we cannot comprehensively understand the connecting mechanisms using only 64-MDCT. To our knowledge, this is the first report to show the details of the connections between the right upper pulmonary vein (RUPV) thrombi and the right lower pulmonary vein (RLPV) thrombi on the anterior wall of the LA using both TEE and 80-MDCT.

## Case presentation

The patient was a 69-year-old female with hypertension. The patient had no history of IS or chest pain. Physical examination revealed no abnormalities. Electrocardiography revealed a normal sinus rhythm and no ST-T changes. The serum D-dimer level was 0.7 μg/mL (normal: <1.0 μg/mL), the activity of protein S was 84% (normal: 74%-132%), and the activity of protein C was 107% (normal: 64%-135%). Her homocysteine level was 11.4 nmol/mL (normal: 5-15 nmol/mL). Her serum brain natriuretic peptide (BNP) level was 18.8 pg/mL (normal: <18.4 pg/mL). We evaluated the PVTs using TEE and 80-slice multidetector computed tomography (80-MDCT).

**Figure 1:**
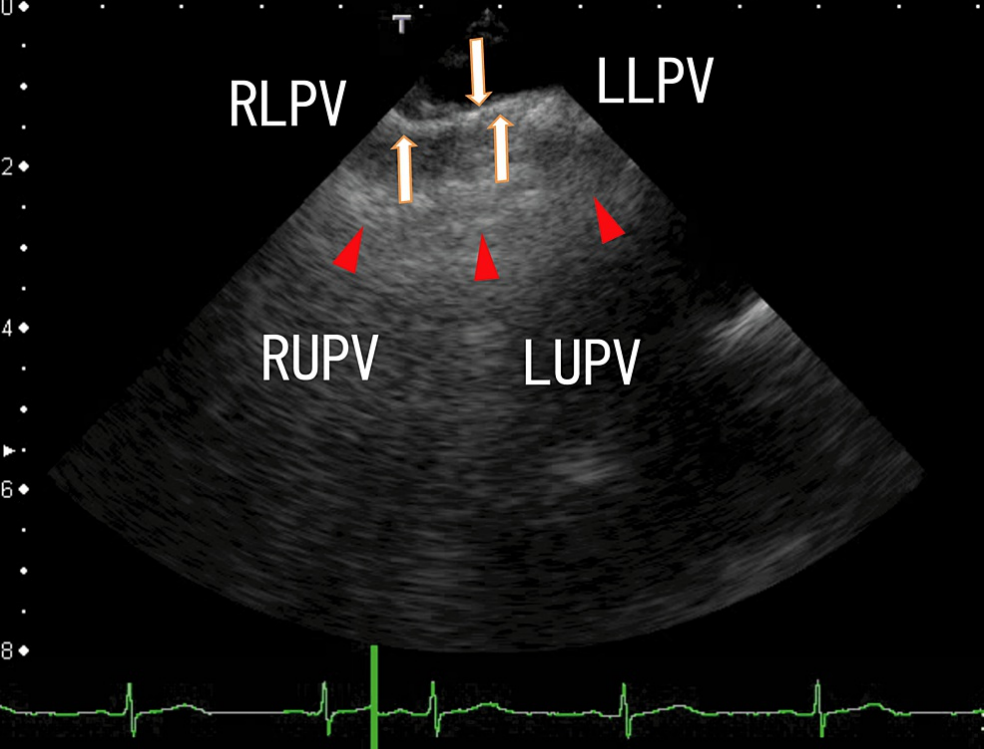
Transesophageal echocardiography (TEE) images showing that the left atrium (LA) thrombi extending from the right lower pulmonary vein (RLPV) thrombi reached the anterior wall of the LA. The arrows indicate the LA thrombi, around which there may be some whitish thrombi. The arrowheads indicate possible walls of the LA. The LA walls had no clear margin; therefore, we hypothesized that there were some thrombi on the LA walls. LLPV, left lower pulmonary vein; LUPV, left upper pulmonary vein; RUPV, right upper pulmonary vein

TEE images showed that the LA thrombi that extended from the RLPV thrombi reached the anterior wall of the LA (Figure 1 and Video 1).

**Video 1:** The video representation of Figure 1. The LA thrombi extending from the RLPV thrombi periodically moved with the heartbeats. The anterior wall of the LA was not smooth, and there were some thrombi, including larger white parts on the anterior wall, which periodically moved with the heartbeats. The LA thrombi extending from the RLPV thrombi had small areas with white shadows, indicating that some of the thrombi were calcified. LA, left atrium; RLPV, right lower pulmonary vein

**Figure 2:**
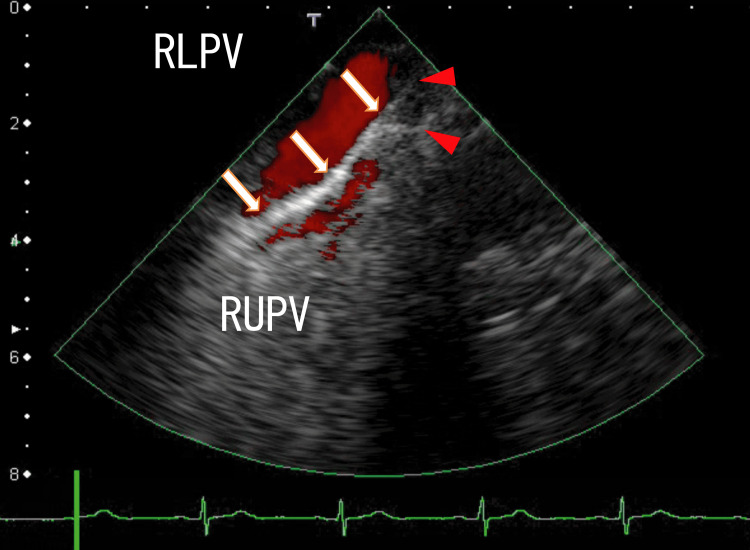
TEE images showed that the LA thrombi extended from the right upper pulmonary vein (RUPV) and reached the anterior wall of the LA. The arrows indicate white LA thrombi, which are connected around the exit of the RUPV and the anterior wall of the LA. The arrowheads indicate the attachment regions of the LA wall, which are near the RLPV thrombus attachment regions. RLPV, right lower pulmonary vein; LA, left atrium; TEE, transesophageal echocardiography

Additionally, TEE images showed that other LA thrombi are connected around the exit of the RUPV and the anterior wall of the LA (Figure 2 and Video 2).

**Video 2:** The video representation of Figure 2. The LA thrombi extending from the RUPV thrombi periodically moved with the heartbeats. The LA thrombi had several small, darker thrombi, which are connected around the thrombi. The LA thrombi had no areas with white shadows. Small red areas sometimes appeared on the left side of the attachment regions, which indicated blood flow from the left upper pulmonary vein (LUPV). LA, left atrium; RUPV, right upper pulmonary vein

**Figure 3:**
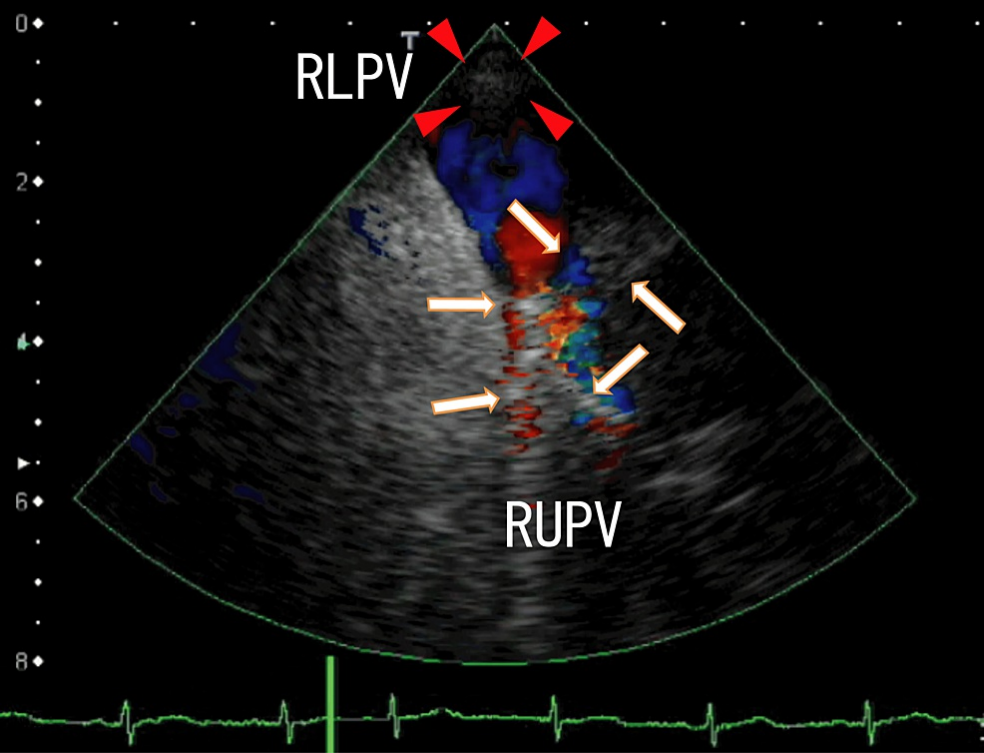
TEE images showing the RUPV thrombi. The arrows indicate the RUPV thrombi, which had two branches. One branch was directed to the left, and the other branch was directed to the right. The LA side of the RUPV thrombi was attached to the left side of the surrounding wall of the exit of the RUPV. In the RUPV, there was a mixture of red, blue, and yellow colors, indicating that there were disturbances in blood flow. The arrowheads indicate the LA thrombi situated around the exit of the RLPV. RLPV, right lower pulmonary vein; RUPV, right upper pulmonary vein; LA, left atrium; TEE, transesophageal echocardiography

Moreover, TEE images showed that the thrombi in the RUPV had two branches: one from the right side and the other from the left side (Figure 3 and Video 3).

**Video 3:** Video representation of Figure 3. The thrombi in the RUPV did not periodically move substantially. The thrombi in the RUPV had no white shadows, but the thrombi had several small, darker thrombi connected to the thrombi. The LA thrombi situated around the exit of the RLPV periodically moved while breathing rather than the heartbeats. LA, left atrium; RUPV, right upper pulmonary vein; RLPV, right lower pulmonary vein

**Figure 4:**
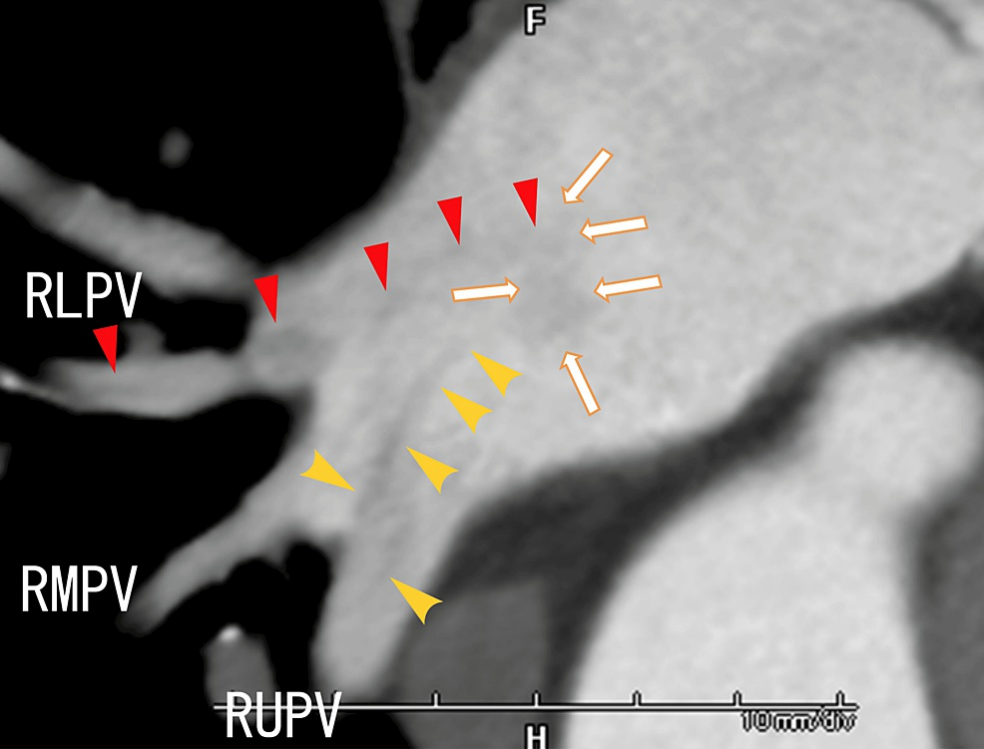
Oblique 80-slice multidetector computed tomography (80-MDCT) images showing thrombi in the right pulmonary veins and the LA. This angle was similar to the angle used for TEE. The yellow arrowheads indicate white LA thrombi extending from the RLPV thrombi, which resemble the white lines depicted using TEE. The red arrowheads indicate the LA thrombi extending from the RUPV thrombi, which resemble white lines with rough surfaces according to TEE. The arrows indicate the attachment areas of both the RUPV thrombi and the RLPV thrombi. There were two right middle pulmonary veins (RMPVs) between the RUPV and RLPV. RLPV, right lower pulmonary vein; RUPV, right upper pulmonary vein; LA, left atrium; TEE, transesophageal echocardiography

Oblique 80-MDCT revealed two LA thrombi extending from the RLPV and RUPV thrombi and attachment regions on the LA wall (Figure 4).

Two thrombi in the LA that extended from the RUPV and RLPV were rather clearly demonstrated using 80-MDCT. The RLPV and RUPV thrombi extended into the LA and reached the nearby region of the LA anterior wall, which was situated on the left-side anterior wall of the exit of the LUPV. The two thrombi looked to be combined on the LA wall, because they had other thrombi around them, which was depicted using both TEE and 80-MDCT (Figure 1, Figure 2, Figure 4, Video 1, and Video 2).

## Discussion

To our knowledge, this is the first report to show the connection between the RLPV thrombi and the RUPV thrombi on the LA anterior wall using both TEE and 80-MDCT.

In our previous studies, the LA thrombi extending from the RLPV thrombi could not be depicted using 80-MDCT [9]; however, in the present case, 80-MDCT revealed that the LA thrombi extended from the RLPV and RUPV thrombi (Figure 4). Additionally, in the present case, 80-MDCT revealed large thrombi, which were depicted as weakly whitish thrombi formed around white linear thrombi using TEE. The cause of these differences when LA thrombi are depicted in 80-MDCT images is unknown.

To our knowledge, this is the first report to show that on TEE, the RUPV thrombi diverged in the right and left directions (Figure 3). Similarly, using 64-MDCT, we previously reported that thrombi in small vessels became increasingly larger and came into the larger-diameter pulmonary vein near the LA [18]. Then, pulmonary vein thrombi extended into the LA and attached to the LA wall, which was detected using 64-MDCT and transthoracic echocardiography (TTE) [9,19]. In the present case, two narrow pulmonary vein thrombi combined into larger RUPV thrombi, which were first described in detail using TEE (Figure 3).

More closely, we noticed that the white RLPV thrombi had small white parts with white shadows, indicating that the LA thrombi extending from the RLPV thrombi had calcified parts; however, the white RUPV thrombi and the white LA thrombi extending from the RUPV had no parts with white shadows. To our knowledge, these findings are the first to be described. In our previous case report, we showed that the LA thrombi extending from the RLPV thrombi, which are located around the exit of the RUPV, had small particles with shadows [13]. The LA thrombi extending from the RUPV thrombi might have no parts with shadows. More studies are needed to clarify these mechanisms.

When pulmonary infection occurs, NETs are produced from neutrophils to kill pathogens. NETs form arterial thrombi in the pulmonary vein to prevent the spread of pathogens to all organs [20]. Then, the information about the pathogens was closed there locationally. When the same pathogen attacks the other side of the lung, antibodies cannot be produced quickly. All lungs should have information on invading pathogens. Leukocytes on PVTs might be connected to transmit pathogen information to the PVTs; therefore, PVTs need to make networks to respond quickly and correctly. Therefore, the RUPV thrombi and the RLPV thrombi combine to form a scaffold for leukocytes. PVTs are naturally related to infections because of their origin. When we think about infection including COVID-19, we need to examine leukocytes on PVTs. More research is required to clarify these relationships.

## Conclusions

The RLPV and RUPV thrombi extended into the LA and reached the near regions on the anterior wall of the LA. They looked to be linked.

The white linear LA thrombi assessed using TEE were depicted as darker linear areas than the surrounding thrombi when they were depicted using 80-MDCT. We might pay attention to leukocytes on PVTs when we think of any infections.

## References

[1] Almekhlafi MA, Hu WY, Hill MD, Auer RN (2008). Calcification and endothelialization of thrombi in acute stroke. Ann Neurol.

[2] Aspegren O, Staessens S, Vandelanotte S (2022). Unusual histopathological findings in mechanically removed stroke thrombi - a multicenter experience. Front Neurol.

[3] Saghamanesh S, Dumitriu LaGrange D, Reymond P, Wanke I, Lövblad KO, Neels A, Zboray R (2022). Non contrast enhanced volumetric histology of blood clots through high resolution propagation-based X-ray microtomography. Sci Rep.

[4] Mak G, Lu JQ, Perera K (2021). Histopathologic analysis of retrieved cerebral thrombi in acute ischemic stroke patients with proximal anterior circulation occlusions amenable to endovascular thrombectomy. J Neurol Sci.

[5] Takeuchi H (2022). Retracted: poster no. 119 large arterial thrombi in the pulmonary vein are common in elderly subjects and may cause age-related disease by producing neutrophil extracellular traps. Cardiovasc Res.

[6] Takeuchi H (2015). Nearly all left atrial thrombi may be extended from pulmonary vein thrombi. Int J Cardiol Heart Vasc.

[7] Sonobe S, Yoshida M, Niizuma K, Tominaga T (2019). Mechanical thrombectomy for acute ischemic stroke arising from thrombus of the left superior pulmonary vein stump after left pneumonectomy: a case report. NMC Case Rep J.

[8] Tsuji Y, Yagi R, Hiramatsu R, Wanibuchi M (2022). Mechanical thrombectomy for acute ischemic stroke due to thrombus in the pulmonary vein stump after left pulmonary lobectomy: a case series. Neurointervention.

[9] Takeuchi H (2017). Rivaroxaban lessens the number of thrombi in the left atrium and right lower pulmonary vein, as illustrated by transoesophageal echocardiography but not 80-MDCT. BMJ Case Rep.

[10] Takeuchi H (2024). The standard-dose heparin-warfarin remedy partially resolves thrombi in the right superior pulmonary vein and left atrium and ameliorates type 2 diabetes mellitus. Cureus.

[11] Takeuchi H (2024). Left atrial diverticula supplied by the anomalistic branch of the right coronary artery. Cureus.

[12] Takeuchi H (2024). The desirable effects of edoxaban on thrombi in the left atrium are seemingly connected to pulmonary vein thrombi. Cureus.

[13] Takeuchi H (2024). Left atrial diverticula present in the right lower pulmonary vein thrombus attachment area. Cureus.

[14] Wu Y, Wei S, Wu X, Li Y, Han X (2023). Neutrophil extracellular traps in acute coronary syndrome. J Inflamm (Lond).

[15] Zhao M, Zheng Z, Yin Z (2023). DEL-1 deficiency aggravates pressure overload-induced heart failure by promoting neutrophil infiltration and neutrophil extracellular traps formation. Biochem Pharmacol.

[16] Bryk AH, Prior SM, Plens K (2019). Predictors of neutrophil extracellular traps markers in type 2 diabetes mellitus: associations with a prothrombotic state and hypofibrinolysis. Cardiovasc Diabetol.

[17] Takeuchi H (2015). A network of pulmonary vein thrombi is a risk factor for ischemic stroke, especially after cardiac surgery: a case report and mini review. Int J Cardiol Heart Vasc.

[18] Takeuchi H (2015). A long and narrow pulmonary vein thrombus attached to the wall of a pulmonary vein. Int J Cardiol Heart Vasc.

[19] Takeuchi H (2015). A jumping left atrial thrombus connected to a pulmonary vein thrombus using transthoracic echocardiography and 64-slice multi-detector computed tomography. Int J Cardiol Heart Vasc.

[20] Fuchs TA, Brill A, Duerschmied D (2010). Extracellular DNA traps promote thrombosis. Proc Natl Acad Sci U S A.

